# Pulmonary hypertension, inhaled nitric oxide, and retinopathy of prematurity: evidence from the U.S. national database

**DOI:** 10.1038/s41390-025-04323-3

**Published:** 2025-08-09

**Authors:** Seohyun Cho, Mohsen A. A. Farghaly, Stacey Ramey, Alshimaa Abdalla, Mohamed A. Mohamed, Hany Aly

**Affiliations:** Neonatology Division, Cleveland Clinic Children’s, Cleveland, OH USA

## Abstract

**Background:**

The study aimed to examine the association of pulmonary hypertension (PH) and the use of inhaled nitric oxide (iNO) with retinopathy of prematurity (ROP) in very low birth weight (VLBW) infants.

**Methods:**

Utilizing the United States National Inpatient Sample (NIS) database, VLBW infants were identified. Chi-square and Fisher’s exact tests were used to calculate unadjusted odds ratios (ORs) for the presence of ROP in relation to PH and iNO therapy. Adjusted odds ratios (aORs) were calculated using logistic regression while controlling for potential confounders.

**Results:**

Among 1,007,141 infants, 30,401 (3%) had PH. ROP was diagnosed more frequently with PH (22.3% vs 13.6%; aOR: 1.32, 95% CI: 1.28–1.36, *p* < 0.001). Severe ROP was less frequent in iNO-treated PH infants (4.33% vs 5.35%; aOR: 0.82, 95% CI: 0.70–0.96, *p* = 0.02). In extremely low birth weight (ELBW) infants with PH, iNO use was associated with lower rates of severe ROP (aOR: 0.68, 95% CI: 0.57–0.81, *p* < 0.001).

**Conclusions:**

PH is associated with an increased risk of ROP. iNO therapy is associated with a decreased prevalence of severe ROP in PH-affected infants. Further studies are needed to assess the early use of vascular modulators in mitigating the risk for ROP.

**Impact:**

- Premature infants diagnosed with pulmonary hypertension are at increased risk for ROP. Severe ROP is less encountered when inhaled nitric oxide was used in this vulnerable population.
- This is the first study to assess the interaction between pulmonary hypertension, its treatment, and the development of ROP in premature infants.
- The study suggests the need for future studies to assess the efficacy of early use of vascular modulators in mitigating the risk of ROP.

## Introduction

Retinopathy of prematurity (ROP) is a proliferative retinal vascular condition that predominantly affects premature infants and is a leading cause of childhood blindness.^1^ The prevalence of ROP is increasing, with 10% of cases classified as severe.^2,3^ Preterm infants requiring higher FiO_2_ concentrations are at a particularly elevated risk of developing ROP, and fluctuations in oxygen supplementation further increase the likelihood of severe ROP.^4^

Pulmonary hypertension (PH), although initially described in term infants, is a serious condition that can also affect preterm infants.^5,6^ Infants with PH often experience wide fluctuations in O_2_ saturation and are typically managed with high FiO_2_ concentrations, both of which contribute to the pathogenesis of ROP.^4,7^ Inhaled nitric oxide (iNO) is widely accepted as the first-line treatment for PH.^5,8^

The interaction between O_2_ therapy, NO, and vascular growth is well documented. Oxygen supplementation acts as a potent pulmonary vasodilator by stimulating nitric oxide (NO) synthase, leading to the release of endothelial NO. Inhaled NO improves both pulmonary and systemic oxygenation by directly dilating the pulmonary vasculature. Additionally, NO interacts with endothelin, a highly effective vasoconstrictor, to control vascular activity and blood circulation related to vascular growth.^1,9^ In a mouse model of retinopathy, reducing NO production in retinal endothelial cells decreased abnormal blood vessel growth and reduced vascular hyperpermeability.^10^ Conversely, reduced endogenous NO expression has been associated with the formation of highly reactive nitrogen species, which directly contribute to microvascular degeneration.^11^ Therefore, hyperoxia contributes to retinal damage, at least in part, by inhibiting endogenous NO synthesis.^1^ However, the relationship between exogenous iNO and retinal development has not been described.

There is very limited data on the association between ROP and PH, and to our knowledge, no studies have specifically investigated the association between iNO and ROP. We hypothesized that preterm infants with PH are at increased risk of developing ROP. Furthermore, because iNO is expected to improve oxygenation, we anticipated that it would reduce endogenous NO production and subsequently decrease the risk of ROP. In this study, we utilized NIS data to assess the relationship between PH and the prevalence of ROP in VLBW infants, as well as the association between iNO treatment and ROP development.

## Methods

This epidemiologic study utilized the NIS dataset from January 1, 2003, to December 31, 2020. The specific aims were: (1) to evaluate the relationship between the occurrence of PH and the development of ROP in VLBW infants and (2) to examine the relationship between iNO treatment and the occurrence of ROP.

### Data source

The NIS dataset is produced by the Healthcare Cost and Utilization Project (HCUP) sponsored by the Agency for Healthcare Research and Quality (AHRQ). NIS is a de-identified, publicly available inpatient healthcare database that collects data on over 7 million hospital stays each year from most states in the United States. NIS uses a stratified, single-stage cluster sampling design, with strata based on region, urban or rural location, teaching status, ownership, and bed size. After stratification, a random sample comprising 20% of hospitals from the target population is included. The HCUP program requires the use of weighted samples to reflect national trends. NIS contains a wide range of information on hospitalized patients, including demographic data, primary and secondary diagnoses, primary and secondary procedures, hospital characteristics, payment source, length of hospital stay, and patient disposition. All types of admissions are included, whether direct admissions, admissions from the emergency room, or transfers from other hospitals.

### Patient selection

Newborn infants were identified in the dataset using the code (Age_Neonate = 1) that is unique to neonatal hospitalization at birth and within the first 28 days of life. Infants admitted after 28 days were not included to avoid the inclusion of infants with chronic PH. In addition, the International Classification of Diseases (ICD) codes were used to identify and categorize infants based on gestational age (GA) and birth weight (BW). The ICD-9 version was applied for the years 2003-2015, while the ICD-10 version was used for 2016-2020. Infants born to mothers with specific medical or perinatal diagnosis were identified using the corresponding ICD codes for newborns affected by maternal conditions. Similarly, infants diagnosed with congenital anomalies, postnatal conditions, or adverse effects were identified using the appropriate ICD codes. For this study, VLBW infants diagnosed with PH were identified using ICD-9 codes 747.83 and 416.8, as well as ICD-10 codes P29.3, P29.30, P29.38, I27.0, I27.2, I27.20, I27.21, and I27.29. Infants who received iNO were identified using ICD-9 procedure code 00.12 and ICD-10 procedure code 3E0F7SD. Since this study utilized a publicly available, de-identified dataset, it was exempt from Institutional Review Board review. Severe ROP was defined as any ROP greater than stage 2, including any ROP that required treatment. The ICD-9 and ICD-10 codes for any ROP and for severe ROP are listed in Supplementary 1.

### Study design

Infants included in the study were divided into two groups: infants with PH and infants without PH. Demographic, perinatal, and clinical characteristics were compared between both groups. The odds ratios (OR) for developing ROP in infants with PH compared to those without PH were calculated using chi-square and Fisher’s exact tests. Furthermore, the occurrence of ROP was compared between infants who received iNO and those who did not, both in the overall population and within the PH group. The association was further analyzed using logistic regression models to calculate adjusted ORs while controlling for confounding variables that may be independently associated with the ROP. Confounding factors included maternal conditions (diabetes, hypertension, or chorioamnionitis), perinatal complications (placenta previa, placental abruption, nuchal cord or cord prolapse), infant demographics (sex, GA, BW, multiple gestation or small for gestational age (SGA) status), congenital anomalies (central nervous system (CNS) and lung anomalies, congenital heart disease (CHD), diaphragmatic hernia (CDH), abdominal wall defects, and common genetic and chromosomal disorders) and postnatal conditions (severe intraventricular hemorrhage (IVH), neonatal seizures, respiratory distress syndrome (RDS), pneumothorax, meconium aspiration, pulmonary hemorrhage, apnea or anemia of prematurity, acute kidney injury (AKI), and bloodstream infection).

### Statistical analysis

Binomial and categorical variables were described using frequencies and percentages. Chi-square and Fisher’s exact tests were used to compare maternal, perinatal, and neonatal characteristics between the two groups. Statistical significance was set at *p* < 0.05. Logistic regression analysis was conducted to assess significant associations while controlling for confounding variables. Data analysis was performed using SAS version 9.4 (SAS Institute Inc., Cary, NC).

## Results

**Fig. 1:**
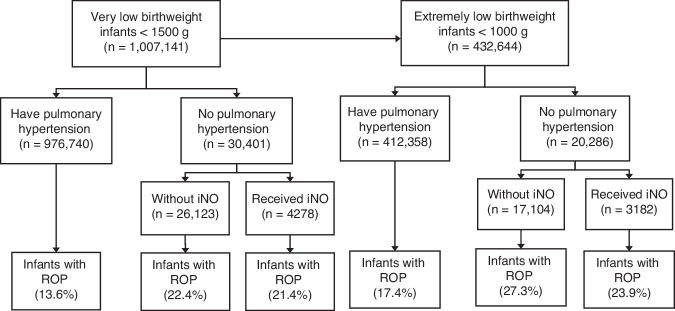
Flow chart for the study population. iNO Inhaled nitric oxide; ROP Retinopathy of prematurity.

The sample included 1,007,141 VLBW infants hospitalized between January 1, 2003, and December 31, 2020 (Fig. 1). In this sample, 49.3% were female infants, 34.5% were Caucasians, 75.5% were singleton pregnancies, and 66.1% were delivered via Cesarean sections. Additionally, 4.3% of infants were classified as SGA, 3% were diagnosed with PH, and 0.42% received iNO therapy. The prevalence of any stage of ROP was 13.8%, while severe ROP occurred in 1.6% of infants.

Maternal, perinatal, congenital, and clinical characteristics were compared between infants diagnosed with PH and those who did not develop PH. Infants with PH had a higher prevalence of male sex, maternal chorioamnionitis, placenta previa, placental abruption, or nuchal cord at delivery. They were also more likely to have a birth weight <1000 g, gestational age <28 weeks, or conditions such as CNS or lung anomalies, CHD, CDH, chromosomal and genetic disorders, hypoxic ischemic encephalopathy, meconium aspiration, pulmonary hemorrhage, sepsis, renal failure, seizures, or severe IVH. Table 1 presents the association (and aOR) of different demographic and clinical variables with ROP and severe ROP.

**Table 1:** Demographic and clinical characteristics of the study population.

	Infants with pulmonary hypertension *n* = 30,401	Infants without pulmonary hypertension *n* = 976,740	Adjusted odds ratios (95% confidence Intervals) and *p* value
Maternal hypertension	2.66	3.25	0.96 (0.94–1.09), 0.78
Maternal diabetes mellitus	2.53	2.34	1.09 (1.01–1.17), 0.03
Chorioamnionitis	2.76	1.70	1.08 (1.04–1.31), 0.05
Placental abruption	1.99	1.62	1.11 (1.02–1.21), 0.02
Female sex	46.2	49.4	0.95 (0.93–0.97), <0.001
Race			
Caucasians	35.5	34.5	Reference
African Americans	24.0	23.0	1.01 (0.96–1.04), 0.10
Hispanic/Latinos	14.9	15.3	0.84 (0.81–0.87), <0.001
Asian/Pacific Islanders	3.58	3.13	1.12 (1.05–1.19), 0.005
Native Americans	0.85	0.68	1.38 (1.21–1.57), <0.001
GA < 28	66.2	48.3	1.62 (1.57–1.66), <0.001
Small for gestational age	1.74	4.36	0.68 (0.63–0.75), <0.001
Nervous system anomalies	4.92	2.40	1.43 (1.35–1.52), <0.001
Lung anomalies	8.48	0.76	8.92 (8.46–9.40), <0.001
Congenital heart disease	26.4	11.5	2.35 (2.29–2.24), <0.001
Diaphragmatic hernia	0.97	0.13	1.41 (1.22–1.64), <0.001
Abdominal wall defects	0.27	0.19	1.12 (0.88–1.41), 0.36
Hypoxic ischemic insults	0.21	0.07	2.25 (1.56–2.67), <0.001
Chromosomal disorders	2.15	0.64	3.25 (2.97–3.56), <0.001
Pneumothorax	18.4	4.50	2.95 (2.85–3.05), <0.001
Pulmonary hemorrhage	5.69	1.66	2.04 (1.93–2.15), <0.001
Apnea of prematurity	60.0	59.5	0.80 (0.78–0.82), <0.001
Severe Anemia	62.4	44.2	1.78 (1.74–1.83), <0.001
Congenital/Acquired Sepsis	48.5	25.8	2.04 (1.99–2.09), <0.001
Severe IVH	14.1	4.83	1.81 (1.74–1.86), <0.001

Data are presented as percentages. Odds ratios are adjusted for the characteristics in the table.

**Fig. 2:**
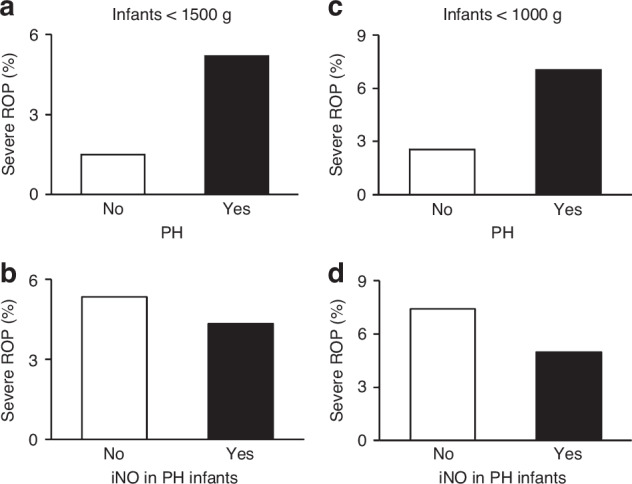
Pulmonary hypertension, inhaled nitric oxide, and severe ROP. Graphs on the left column are for <1500 g infants whereas graphs on the right column represent infants <1000 g. Panels (**a**, **c**) represent the relationship between pulmonary hypertension and severe ROP. Panels (**b**, **d**) represent the relationship between inhaled nitric oxide and severe ROP in infants with pulmonary hypertension. Severe ROP was significantly increased with PH, and in those with PH, the use of iNO was associated with significantly decreased severe ROP. PH Pulmonary hypertension, iNO Inhaled nitric oxide, ROP Retinopathy of prematurity.

Among infants diagnosed with PH, 22.3% developed any stage of ROP, compared to 13.6% in those not diagnosed with PH (aOR: 1.32, 95% CI: 1.28–1.36, *p* < 0.001). The prevalence of severe ROP was 5.21% in infants with PH compared to 1.48% in those without PH (aOR: 2.24, 95% CI: 2.12–2.37, *p* < 0.001) (Fig. 2). In the overall sample, the prevalence of ROP among infants who received iNO therapy compared to those who did not was 21.4% and 13.8%, respectively (aOR: 1.31, 95% CI: 1.21-1.41, *p* < 0.001) (Table 2 and Fig. 2). Of note, among infants diagnosed with PH, the prevalence of ROP was 21.4% in those who received iNO compared to 22.4% in those who did not, with no statistical difference (aOR: 1.03, 95% CI: 0.95–1.12, *p* = 0.53). However, the prevalence of severe ROP among those with PH who received iNO versus those who did not was 4.33% and 5.35%, respectively, showing a statistically significant difference (aOR: 0.82, 95% CI: 0.70–0.96, *p* = 0.02) (Table 2).

**Table 2:** Retinopathy of prematurity, pulmonary hypertension, and inhaled nitric oxide in infants <1500 g.

	Number	ROP	aOR (95% CI)	*p* value	Severe ROP	aOR (95% CI)	*p* value
Pulmonary hypertension	No (976,740)	13.6	1.32 (1.28–1.36)	<0.001	1.48	2.24 (2.12–2.37)	<0.001
	Yes (30,401)	22.3			5.21		
Inhaled nitric oxide	No (1,002,863)	13.8	1.31 (1.21–1.41)	<0.001	1.59	1.69 (1.45–1.96)	<0.001
	Yes (4278)	21.4			4.33		
Use of inhaled nitric oxide in infants with PHN	No (26,123)	22.4	1.03 (0.95–1.12)	0.53	5.35	0.82 (0.70–0.96)	0.02
	Yes (4278)	21.4			4.33		

Regression analysis was performed while controlling for maternal chorioamnionitis, perinatal factors (placenta previa and cord prolapse), demographic characteristics (sex and small for gestational age), presence of congenital anomalies (central nervous system, lung, or heart, anomalies, diaphragmatic hernia, abdominal wall defects, and common chromosomal and congenital disorders), and clinical confounders that may interpedently contribute to the development of PHN (pneumothorax, pulmonary hemorrhage, apnea or anemia of prematurity, neonatal sepsis or seizures, and severe intraventricular hemorrhage.

*aOR* adjusted odds ratios, *PHN* pulmonary hypertension, *ROP* retinopathy of prematurity.

ELBW infants followed a similar trend when comparing those diagnosed with PH or treated with iNO to those without PH or iNO treatment (Table 3). Furthermore, among infants with PH, those who received iNO had a significant reduction in severe ROP compared to those who did not (aOR: 0.68, 95% CI: 0.57–0.81, *p* < 0.001).

**Table 3:** Retinopathy of prematurity, pulmonary hypertension, and inhaled nitric oxide in infants <1000 g.

	Number	ROP	aOR (95% CI)	*p* value	Severe ROP	aOR (95% CI)	*p* value
Pulmonary hypertension	No (412,358)	17.4	1.25 (1.21–1.29)	<0.001	2.53	2.03 (1.92–2.16)	<0.001
	Yes (20,286)	26.7			7.03		
Inhaled nitric oxide	No (429,462)	17.8	1.20 (1.10–1.31)	<0.001	2.73	1.34 (1.14–1.58)	<0.001
	Yes (3182)	23.9			4.96		
Use of inhaled nitric oxide in infants with PHN	No (17,104)	27.3	0.95 (0.87–1.04)	0.29	7.42	0.68 (0.57– 0.81)	<0.001
	Yes (3182)	23.9			4.96		

Regression analysis was performed while controlling for maternal chorioamnionitis, perinatal factors (placenta previa and cord prolapse), demographic characteristics (sex and small for gestational age), presence of congenital anomalies (central nervous system, lung, or heart, anomalies, diaphragmatic hernia, abdominal wall defects, and common chromosomal and congenital disorders), and clinical confounders that may interpedently contribute to the development of PHN (pneumothorax, pulmonary hemorrhage, apnea or anemia of prematurity, neonatal sepsis or seizures, and severe intraventricular hemorrhage.

*aOR* adjusted odds ratios, *PHN* pulmonary hypertension, *ROP* retinopathy of prematurity.

Sensitivity analyses were repeated after excluding infants with congenital malformation and genetic disorders. In VLBW infants diagnosed with PH, the use of iNO was associated with aOR of 1.03, 95% CI: 0.93–1.15, *p* = 0.52 for ROP and aOR of 0.91, 95% CI: 0.75–1.11, *p* = 0.36 for severe ROP. In ELBW infants diagnosed with PH, the use of iNO was associated with aOR of 0.95, 95% CI: 0.85–1.06, *p* = 0.35 for ROP and aOR of 0.74, 95% CI: 0.60–0.92, *p* = 0.006 for severe ROP.

Table 4 presents other factors that were significantly associated with the development of severe ROP, including chorioamnionitis, cord prolapse, CNS anomalies, CHD, RDS, pneumothorax, MAS, apnea of prematurity, anemia of prematurity, bloodstream infections, acute renal failure, seizures, and severe IVH.

**Table 4:** Factors associated with the development of severe retinopathy of prematurity in very low birth weight infants.

	Adjusted odds ratio of confounding factors associated with retinopathy of prematurity in very low birth weight infants
Gestational age <28 weeks	2.17 (2.14–2.20), <0.001
CNS anomalies	1.41 (1.36–1.46), <0.001
Congenital heart disease	1.40 (1.38–1.42), <0.001
Congenital diaphragmatic hernia	1.43 (1.22–1.670, <0.001
Apnea of prematurity	1.90 (1.88–1.93), <0.001
Anemia of prematurity	2.90 (2.87–2.95), <0.001
Bloodstream infection	1.16 (1.15–1.18), <0.001

Adjusted odds ratios (aOR) calculated comparing between the two groups while controlling for maternal chorioamnionitis, perinatal factors (placenta previa and cord prolapse), demographic characteristics (sex and small for gestational age), presence of congenital anomalies (central nervous system, lung, or heart, anomalies, diaphragmatic hernia, abdominal wall defects, and common chromosomal and congenital disorders), and clinical confounders that may interpedently contribute to the development of PHN (pneumothorax, pulmonary hemorrhage, apnea or anemia of prematurity, neonatal sepsis or seizures, and severe intraventricular hemorrhage.

## Discussion

This study demonstrated that PH is associated with an increased risk of ROP and severe ROP in premature infants. It is the first to demonstrate that the use of iNO is associated with a significant reduction in severe ROP among premature infants diagnosed with PH.

Infants with PH exhibited higher rates of ROP and severe ROP compared to those without PH. This association may be attributed to the overall poorer health and clinical instability of infants with PH, who often also present with additional factors that increase the risk of ROP. These infants typically require higher oxygen supplementation, exposing them to greater oxidative stress.^1,9,12^ Furthermore, they experience more frequent fluctuations in oxygen saturation, which increases the risk of severe ROP.^4^ Inflammation plays a key role in the development of PH.^5,6,13–15^ Inflammation has also been shown to significantly contribute to development of ROP. Various inflammatory factors can directly affect retinal neovascularization, including cytokines, NO, and hypoxia-inducible factors.^16^ Notably, systemic inflammation, which can be triggered by various conditions such as sepsis, may lead to cardiopulmonary compromise, altering retinal blood flow.^17^ Infants with PH are also at increased risk of prolonged ventilatory support, which has been identified as a major risk factor for ROP.^7^ A study examining the relationship between cardiovascular diseases and ROP found that infants with PH were more likely to develop severe ROP, though no significant association was observed with milder forms of ROP. Nonetheless, potential misclassification of complex cardiovascular cases may have affected the results.^18^

This study demonstrated that chorioamnionitis is a contributing factor to the increased prevalence of PH. A previous report revealed that preterm infants exposed to chorioamnionitis experienced negative pulmonary sequelae, including PH.^19^ Another study found that inducing chorioamnionitis prenatally in preterm lambs triggered an inflammatory response, resulting in vascular changes in the lungs typical of PH.^20^ In addition to prenatal exposure to infection, postnatal sepsis was also associated with an increased risk of PH. Sepsis is a known risk factor associated with PH.^5,6,13,14^ In a study using echocardiography to diagnose PH, 34.4% of neonates with sepsis were identified as having PH. Although the exact pathophysiology is not fully understood, one potential explanation is that neonates with sepsis frequently experience respiratory failure, potentially leading to constriction of pulmonary blood vessels due to hypoxia. These infants are also more likely to require increased ventilatory support, which can further contribute to pulmonary vasoconstriction.^15^ Additionally, pneumonia or sepsis caused by bacterial infection can lead to the release of bacterial endotoxins, triggering an inflammatory cascade that results in PH.^13^

Inhaled NO was associated with decreased severe ROP in preterm infants with PH. Nitric oxide can exert both cytotoxic and cytoprotective effects, depending on the redox state.^21^ During oxidative stress, the activity of eNOS is altered, thereby generating more superoxides by uncoupling oxygen reduction from NO production.^1,21^ In this state, the availability of NO to interact with reactive oxygen species is diminished, promoting the formation of highly reactive nitrogen species that contribute to microvascular degeneration.^1,11,21^ Furthermore, the presence of reactive oxygen species leads to consumption of endogenous antioxidants, consequently depleting the antioxidative reserve. Conversely, in a non-oxidative environment, NO acts as a cytoprotective agent, which potentially increases the expression of vascular endothelial growth factor receptor-2, which is essential for the survival of newly formed retinal vessels. Endothelial NO synthase, an isovariant of NO synthase, plays an important role in retinal angiogenesis. Following several days of treatment with high oxygen and an NO synthase inhibitor, both endothelial NO synthase and superoxide dismutase activity were elevated. Superoxide dismutase, known for its antioxidative properties, helps reduce superoxide anions and balance the redox state. This subsequently promotes an increase in vascular endothelial growth factor receptor-2 expression, which is essential for blood vessel formation and endothelial cell survival.^8,21^ Furthermore, a study has shown that iNO improved the redox state in very preterm infants during the early days of life, leading to a decreased utilization of antioxidants from endogenous reservoirs, thereby reducing oxidative stress.^8^ It can be assumed that many infants with PH who require iNO receive the treatment earlier, within the first few days of life. Early treatment may result in a more rapid improvement in respiratory failure, leading to a reduced need for high levels of FiO_2_ in the first few days of life and minimizing fluctuations in FiO_2_ exposure. This reduction in oxygen variability may help decrease retinal damage caused by reactive oxygen species. This could explain why ELBW infants, who are generally more critically ill and receive iNO earlier, exhibited a significant reduction in severe ROP compared to the VLBW infants.

This study demonstrated an association between anemia and severe ROP. While some studies have identified a positive correlation between anemia and the development of ROP, others have not supported such finding.^22^ Despite this inconsistency, several studies in the literature have reported an association between the number of blood transfusions and an increased risk of severe ROP in preterm infants.^22^ One proposed mechanism underlying this association involves the elevation of non-transferrin-bound iron following transfusion in preterm infants, which can promote the production of reactive oxygen species.^23^ Of note, transfused blood is composed of adult red cells that have significantly less oxygen affinity than neonatal blood, thereby releasing oxygen to tissues, including the retina, much faster than physiologically expected.

The apnea of prematurity was also associated with severe ROP. Prior research has identified apnea as an independent risk factor for the development of stage 2 or greater ROP.^24^ Moreover, increased episodes of intermittent hypoxemia have been independently linked to cases of ROP regardless of gestational age and severity of illness.^25^ Infants with apnea of prematurity are more likely to experience greater fluctuations in oxygen supplementation and require mechanical ventilation, both of which are known contributors to the pathogenesis of ROP.

Intraventricular hemorrhage (IVH) was associated with severe ROP. This relationship has been observed in previous studies, which have reported IVH as a potential risk factor for ROP.^7,26^ This association may be attributed to the presence of common underlying mechanisms. Both conditions arise in part from the fragility of underdeveloped blood vessels in the brains and retinas of preterm infants. Furthermore, inconsistent blood flow and fluctuation in oxygen can lead to oxidative stress, creating conditions that promote hypoxic injuries.

This study has multiple strengths. To the best of our knowledge, it is the first to investigate the association between iNO and ROP. Additionally, it further explores the relationship between PH and ROP, an area that has been understudied. The study benefits from a large sample size of over one million neonates from across the United States, providing a robust and accurate analysis of the association among ROP, PH, and iNO. However, the study inherited some limitations, primarily related to the use of ICD codes. Although the NIS maintains rigorous standards to ensure dataset accuracy, the study cannot confirm that all PH diagnoses and iNO use were consistently reported by providers. Furthermore, the dataset does not specify whether PH diagnoses were made clinically or via echocardiography. Another potential limitation is that some neonates may have had transient PH that did not require iNO. Therefore, the potential for misclassification bias could have been encountered. The study did not have long-term follow-up for infants with and without PH, though such outcomes were not within the intended study design. Although the study utilized large sample size representing the entire U.S., which can be powerful, it relied solely on administrative data, using ICD codes to define both exposure and outcome variables. We applied proper methodological safeguards; however, cautious interpretation of the current results should be exercised.

In conclusion, PH is independently associated with an increased risk of ROP in VLBW and ELBW infants. iNO therapy is associated with a reduced prevalence of severe ROP in infants diagnosed with PH. While iNO is not a treatment for ROP, these findings suggest it may have a protective effect against severe ROP in the context of PH. Future prospective studies are needed to evaluate the efficacy and optimal dosing of iNO in mitigating the risk of ROP in infants with PH.

## Supplementary information

Supplement 1
