# Supplementary material for: Pulmonary hypertension, inhaled nitric oxide, and retinopathy of prematurity: evidence from the U.S. national database
Source: Pediatr Res. 2025 Aug 9;99(3):915–20. doi: 10.1038/s41390-025-04323-3 (PMC13021490; doi:10.1038/s41390-025-04323-3)
Supplement: Supplementary file 1 — Supplement 1 [file 41390_2025_4323_MOESM1_ESM.pdf]

International Classification of Diseases (ICD) codes 9 and 10 used for ROP.

| **Diagnosis** | **ICD 9 Codes** | **ICD 10 Codes** |
| --- | --- | --- |
| Retinopathy of Prematurity Unspecified | 36.210<br>36.220 | H35.101<br>H35.102<br>H35.103<br>H35.109 |
| Retinopathy of Prematurity Stage 0 | 36.222 | H35.111<br>H35.112<br>H35.113<br>H35.119 |
| Retinopathy of Prematurity Stage 1 | 36.223 | H35.121<br>H35.122<br>H35.123<br>H35.129 |
| Retinopathy of Prematurity Stage 2 | 36.224 | H35.131<br>H35.132<br>H35.133<br>H35.139 |
| Retinopathy of Prematurity Stage 3 | 36.225 | H35.141<br>H35.142<br>H35.143<br>H35.149 |
| Retinopathy of Prematurity Stage 4 | 36.226 | H35.151<br>H35.152<br>H35.153<br>H35.159 |
| Retinopathy of Prematurity Stage 5 | 36.227 | H35.161<br>H35.162<br>H35.163<br>H35.164 |
